# Severe Allergic Bronchopulmonary Mycosis and Long-Term Follow-Up

**DOI:** 10.1155/2018/4251673

**Published:** 2018-08-12

**Authors:** Hossein Esmaeilzadeh, Sara Kashef, Hamid Reza Hatami, Soheila Alyasin, Hesamodin Nabavizadeh, Elmira Esmaeilzadeh

**Affiliations:** ^1^Allergy research center, Shiraz University of Medical Sciences, Shiraz, Iran; ^2^Department of Allergy and Clinical Immunology, Namazi Hospital, Shiraz University of Medical Sciences, shiraz, Iran; ^3^Department of Internal Medicine, Division of Rheumatology, Shiraz University of Medical Sciences, Shiraz, Iran

## Abstract

Allergic bronchopulmonary aspergillosis (ABPA) is the most common immunologic reaction following fungal allergen exposure in asthmatic patients. A less frequent syndrome in response to other fungal species like candida is allergic bronchopulmonary mycosis (ABPM). This reaction is mostly associated with asthma exacerbation, changes in Immunoglobulin E levels, and nonspecific findings in high resolution computed tomography (HRCT). This study presents a 9-year-old girl, a known case of childhood asthma, resolved 4 years ago as a novel case of ABPM due to* Candida albicans* manifested by severe emphysema, bronchiectasis, and pneumothorax which consequently required long-term treatment to get relieved.

## 1. Introduction

The exposure of asthmatic patients to indoor and outdoor fungal allergens causes noninvasive severe allergic reactions [1–3]. The most common immunologic reaction is allergic bronchopulmonary aspergillosis (ABPA) and a less frequent syndrome in response to other fungal species is allergic bronchopulmonary mycosis (ABPM) [1]. ABPM is characterized by asthma exacerbation, infiltration in chest radiograph, peripheral blood eosinophilia, high titer total IgE, and immunologic response to fungi other than aspergillus by positive specific IgG and IgE [3, 4]. The findings of chest radiograph were mostly nonspecific; therefore, high resolution computed tomography (HRCT) is considered as the modality of choice for the diagnosis of ABPM. The findings of HRCT include central bronchiectasis and mucus plugging besides bronchocele formation [4, 5]. Other characteristic findings in ABPM are hypersensitivity, inflammation of pulmonary parenchyma, goblet cell metaplasia, and mucus formation [6]. In children, ABPM is mainly caused by* Candida albicans*, Curvularia,* Pseudallescheria boydii*, and Bipolaris [4]. This study presents a 9-year-old girl who had a known case of childhood asthma, resolved 4 years ago, as a novel case of ABPM resulting from* Candida albicans*, manifested by severe emphysema, bronchiectasis, and pneumothorax which required long-term treatment to get relieved.

## 2. Case Presentation

A 9-year-old girl with respiratory distress, dry cough exacerbated at night and triggered by exercise, and fever for about 48 h before admission was admitted to our department. In her past medical history, she was diagnosed of previous childhood asthma at 3 years of age. Atopy history and skin prick test of aeroallergens in past medical history and records were negative. Asthma control was achieved with inhale corticosteroid and asthma treatment stopped after two years. The patient had neither had an asthma attack nor needed asthma related medication in the last 4 years of her life. Latest pulmonary function test was one year before admission, which revealed FEV1: 85%, FEV1/FVC: 91%, FVC: 93%, and PEF: 78%. The initial physical examination revealed diffuse rales and wheezing. Her vitals revealed tachypnea (respiratory rate: 32), tachycardia (pulse rate: 135), temperature of 38, and oxygen saturation levels of 80% in room air. Chest X-ray revealed perihelia infiltration. The patient was hospitalized primarily based on the impression of being plagued with asthma and pneumonia; thus, specific treatment for asthma and antibiotic therapy for pneumonia was initiated. Seventy-two hours later, antibiotics were changed from Clindamycin to Meropenem plus Vancomycin and Azithromycin. The fever subsided in the patient within 48 h and the symptoms of cough and respiratory distress improved significantly. The asthma symptoms were also improved.

The laboratory findings were as follows: white blood cell count of 10700/mL with 1% eosinophils and IgE level of 1075 IU/ml (normal range: 20-100) (Table 1). Chest CT SCAN revealed mild ground glass appearance, 72 hours later. Skin prick test was negative for aspergillosis. Bronchoscopy was carried out and bronchoalveolar lavage (BAL) secretion was analyzed for gram stain and sent for polymerase chain reaction (PCR) to check for aspergillosis, candida, and tuberculosis that all were negative. In BAL Cytometry, the most dominant cell was macrophage (75%) and less than 5% was eosinophil. The patient was discharged after 7 days with 250 micro fluticasone daily inhaler and oral prednisolone 0.5 mg/kg per day (for 2 days more) by diagnosis of asthma relapse.

Four days later, the patient was readmitted with cough, dyspnea, and diffuse bilateral wheeze. The results obtained from the physical examination were similar to previous findings except for the absence of fever. Laboratory tests revealed WBC: 14700/*μ*l with 30% eosinophil. IgE levels were 1359 IU/mL and 1661 IU/mL in double-checking. The results of further laboratory tests are summarized in Table 1.

**Figure 1:**
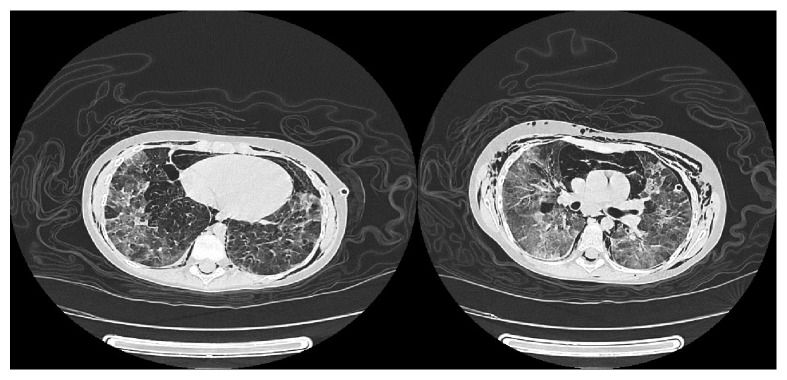
Severe emphysema in chest wall and bronchiectasis in spiral chest CT scan.

On the 2nd day of admission, the patient developed dyspnea and severe subcutaneous emphysema in the anterior and posterior areas of the neck. Spiral chest CT scan revealed severe pneumo-mediastinum and severe emphysema in the chest wall (Figure 1). In addition, ground glass densities and findings in favor of bronchiectasis were also reported in both lungs.

Stool examination was carried out to check for eosinophilia, but the result was negative. According to the high titer of total IgE and eosinophilia, follow-up works were carried out for allergic bronchopulmonary aspergillosis (ABPA), which was negative for specific IgG (18.5mg/ml, cut-off<50) and specific IgE (<0.1IU/ml, cut-off<0/1) of aspergillosis and specific IgG (4.2, ref<113) of Candida but positive for specific IgE (0.74, cut-off <0.1) of Candida. The report of BAL bronchoscopy in previous admissions revealed the presence of* Candida albicans*. The patient was admitted in the intensive care unit (ICU) because of the decrease in breathing sounds and severe respiratory distress. She was once again placed on Meropenem and Vancomycin medication. As a result of progressive emphysema and decreased O_2_ saturation, a chest tube was inserted. Intravenous infusion (IV) of methylprednisolone 1 mg/kg/day plus IV fluconazole 6 mg/kg/day in the first day and following 4 mg/kg/day in the following days was administered. After one week, the chest tube was removed and respiratory distress was improved markedly. The patient was transferred to a ward for final diagnosis of ABPM with a high dose of Itraconazole (200mg twice daily) and high doses of oral prednisolone (0.75 mg/kg per day divided twice daily) and was discharged after 10 days. The same doses of Prednisolone and Itraconazole were continued on the patient using the same doses; and Fluticasone plus Salmetrol inhaler spray (250micro/day divided twice daily) and oral Montelukast were also prescribed for relieving severe asthma attack. Oxygen supplement according to oxygen saturation assay was also recommended. In further follow-up, after one month, the patient's general condition improved significantly and the use of oxygen was no longer necessary. The IgE level decreased to 255 IU/mL and the patient had normal social activity and normal lung sounds. After 2 months, by decreasing prednisolone dose to 25%, asthma symptoms worsened; therefore, uptitration of prednisolone was carried out to reach the previous administered doses. After 3 months, prednisolone was tapered by 25% every four weeks; and after 4 months, the patient stopped receiving prednisolone with good asthma control and IgE level of 86 IU/mL. The total eosinophil count decreased to 100/*μ*l in the peripheral blood sample. After 6 months, asthma medication decreased to 125 Fluticasone per day as the doses were needed for mild persistent asthma. Thus, good asthma control was achieved. After passing 6 months, all drugs were stopped and no other respiratory complaint has been reported in the last 4 months.

## 3. Discussion

Hinson et al. (1952) reported that allergic bronchopulmonary mycosis (ABPM) is a less frequent allergic reaction to fungal allergens in asthmatic patients, compared to allergic bronchopulmonary aspergillosis (ABPA) [1, 2].

In a study by Agrawal et al., the prevalence of ABPA was observed to be higher in patients with acute severe asthma compared to outpatient with bronchial asthma (39% versus 21%) [7, 8].

This study introduced ABPM, which was present in a previous case of childhood asthma. This case was characterized by sudden onset cough and respiratory distress and was found with severe acute emphysema and bronchiectasis in HRCT. In a review, Anuradha Chowdhary et al. [9] in 2012 showed that cough, dyspnea, and asthma-like symptoms are common presentations in ABPM patients as seen in the presented case. Most previous cases diagnosed with ABPM, were in the severe uncontrolled asthma stage [3]; however, the key point of this case was that she had no sign of asthma for the last 4 years. Bhagteshwar Singh et al. reported a 21-year-old patient with ABPM due to Alternaria, who had the same symptoms [10].

Serum IgE levels above 200 IU/mL is a diagnostic tool in ABPM. Therefore, the diagnosis of ABPM was made in our patient after receiving a positive IgE test. Yuma Fukutomi et al. reported that the serum IgE level is one of the definite diagnostic tools for ABPM. This finding was confirmed by other studies [3, 9, 10]. Despite the fact that no study has reported a cut-off value for IgE levels in the diagnosis of ABPA, many researchers have used 1,000IU/mL as cut-off [7]. In our patient, there was a marked increase in the IgE level (1075IU/ml) compared to the laboratory results of the previous 2 years (120IU/ml).

Although a low eosinophil count does not exclude the ABPM, eosinophil counts> 1,000 cells/*μ*L are mostly in favor of this diagnosis, especially, in ABPA [7]. Our reported case had eosinophil count of 3210 cells/*μ*L, which is compatible with the diagnostic criteria for ABPA.

HRCT is the modality of choice for the diagnosis of ABPA and ABPM. The findings include parenchymal lung opacification which may progress to collapse or central bronchiectasis and mucus plugging [11–15]. Pleural thickening was also a common finding in the CT scan of chronic ABPA patients [12]. In complicated severe asthma, bronchiectasis may be present in HRCT; yet, this involvement should not exceed two lobes, as seen in ABPA [16].

The findings of chest CT scan in our patient were in favor of ABPM diagnosis. Pneumothorax, emphysematous changes, bleb, and pulmonary fibrosis are the main radiologic findings in severe ABPA [16, 17]. There is no other classification for ABPM compared to ABPA in literatures. Consequently, it appears that our patient meets the criteria of severe ABPM, considering the radiologic and clinical findings.

Treatment of ABPM was achieved by fulfilling several objectives. The first objective was suppressing immune response to allergens and eradicating fungi colonization in airways [18]. To achieve this objective, high doses of Itraconazole and prednisolone were prescribed. The patient was initially treated with a daily dosage of 0.5mg/kg prednisolone and 100 mg Itraconazol. After confirmation of the diagnosis, the doses were increased to 0.75mg/kg prednisolone and 200 mg twice a day of Itraconazol. Thereafter, after 3 months, we tapered the steroid by 20% every 3 months. Previous studies also confirmed that steroids are fundamental therapies for ABPM [9, 10].

Another clinical goal in the treatment of ABPM was the removal of any bronchial mucus plug and lowering or discontinuing patient's exposure to etiologic fungi [18]. The course of ABPA treatment to stop receiving corticosteroid is mostly 3-4 months [13, 14]; yet, in our study, with severe ABPM, the total treatment course lasted 7 months. Long-term prescription of steroid and additive Itraconazole in our patient helped us in achieving this aim.

Underlying immunodeficiency can cause sudden onset pulmonary involvement and immunologic reactions to fungal or bacterial agents such as common variable immunodeficiency (CVID), chronic granulomatous disease (CGD), hyper-IgE syndrome, and human immunodeficiency virus (HIV) [19, 20]; yet, in our cases immune work ups for antibody and cellular primary and secondary immunodeficiency were negative and there was no sign of immunodeficiency in her history and physical exam.

## Figures and Tables

**Table 1:** Laboratory test results of the patient. (WBC = white blood cell, ESR = erythrocyte sedimentation rate, CRP = C-reactive protein, Ig = immunoglobulin, C = complement, CH50 = hemolytic complement, ANA = antinuclear antibodies, C-ANCA = C-anti neutrophilic cytoplasmic antibody, P-ANCA= P-anti neutrophilic cytoplasmic antibody, HIES = hyper-IgE syndrome.)

**Test**	**First Admission**	**Second Admission**	**Normal Range**
WBC	10700/ *μ*l	14700	3500 -11000/ *μ*l
Neutrophils	8667/ *μ*l	5880/ *μ*l	
Lymphocytes	1498/ *μ*l	4116/ *μ*l	
Eosinophil	107/*μ*l	4410/ *μ*l	
Monocyte	428/ *μ*l	294/ *μ*l	

Hemoglobin	12.2 g/dl	13 g/dl	12-16 g/dl

Platelet	210 x10^∧^9/L	270 x10^∧^9/L	150-450 x10^∧^9/L

ESR	12mm/l	26 mm/l	Up to 20 mm/l

CRP	1mg/l	14 mg/l	<6 mg/l = negative

Albumin	4.3gr %		3/5-5/2 %

IgM	1.55g/l		0/24-2/1 g/l

IgA	2.7 g/l		0/34-3/05 g/l

IgG	8.68 g/l		5/53-13/07 g/l

Anti-Tetanus Antibodies	0.26 IU/ml		>0/1 IU/ml

DHR	198%		>50%

C3	134mg/dl		90-180 mg/dl

C4	29.2 mg/dl		10-40 mg/dl

CH50	116%		>80%

ANA	0.3 u/ml		<10 u/ml

Sweat Chloride Test	45mmol/l	30 mmol/l	<60 mmol/l

Flow Cytometry	CD3: 62% (928/ *μ*l) CD4: 43% (644/ *μ*l) CD8: 16% (239/ *μ*l) CD19: 27% (404/ *μ*l) CD20: 27% (404/ *μ*l) CD16: 11% Interferon Gama receptor: 98% of leukocytes express		50-77% (total T cell) 33-58% (T helper) 13-26% (T cytotoxic) 13-35% (B cell ) 13-35% (B cell) 2-13% (NK cell)

P-ANCA	1.1 u/ml		<12 u/ml

CANCA	2.6 u/ml		<12 u/ml

HIV test	Negative		

HIES score	16		<20 unlikely to indicate Autosomal dominant HIES

Tuberculin PPD test	4 millimeter		> 10 millimeter positive for tuberculosis
